# Systematic review and dosage analysis: hyperbaric oxygen therapy efficacy in the treatment of posttraumatic stress disorder

**DOI:** 10.3389/fneur.2024.1360311

**Published:** 2024-05-31

**Authors:** Susan R. Andrews, Paul G. Harch

**Affiliations:** ^1^Neuropsychological Services for Children and Adults, Metairie, LA, United States; ^2^Section of Emergency and Hyperbaric Medicine, Department of Medicine, LSU Health Sciences Center, New Orleans, LA, United States

**Keywords:** post traumatic stress disorder, hyperbaric oxygen therapy, treatment, trauma, anxiety disorder, PTSD, HBOT

## Abstract

**Background:**

Studies of hyperbaric oxygen therapy (HBOT) treatment of mild traumatic brain injury persistent postconcussion syndrome in military and civilian subjects have shown simultaneous improvement in posttraumatic stress disorder (PTSD) or PTSD symptoms, suggesting that HBOT may be an effective treatment for PTSD. This is a systematic review and dosage analysis of HBOT treatment of patients with PTSD symptoms.

**Methods:**

PubMed, CINAHL, and the Cochrane Systematic Review Database were searched from September 18 to November 23, 2023, for all adult clinical studies published in English on HBOT and PTSD. Randomized trials and studies with symptomatic outcomes were selected for final analysis and analyzed according to the dose of oxygen and barometric pressure on symptom outcomes. Outcome assessment was for statistically significant change and Reliable Change or Clinically Significant Change according to the National Center for PTSD Guidelines. Methodologic quality and bias were determined with the PEDro Scale.

**Results:**

Eight studies were included, all with < 75 subjects/study, total 393 subjects: seven randomized trials and one imaging case-controlled study. Six studies were on military subjects, one on civilian and military subjects, and one on civilians. Subjects were 3-450 months post trauma. Statistically significant symptomatic improvements, as well as Reliable Change or Clinically Significant changes, were achieved for patients treated with 40-60 HBOTS over a wide range of pressures from 1.3 to 2.0 ATA. There was a linear dose-response relationship for increased symptomatic improvement with increasing cumulative oxygen dose from 1002 to 11,400 atmosphere-minutes of oxygen. The greater symptomatic response was accompanied by a greater and severe reversible exacerbation of emotional symptoms at the highest oxygen doses in 30-39% of subjects. Other side effects were transient and minor. In three studies the symptomatic improvements were associated with functional and anatomic brain imaging changes. All 7 randomized trials were found to be of good-highest quality by PEDro scale scoring.

**Discussion:**

In multiple randomized and randomized controlled clinical trials HBOT demonstrated statistically significant symptomatic improvements, Reliable Changes, or Clinically Significant Changes in patients with PTSD symptoms or PTSD over a wide range of pressure and oxygen doses. The highest doses were associated with a severe reversible exacerbation of emotional symptoms in 30-39% of subjects. Symptomatic improvements were supported by correlative functional and microstructural imaging changes in PTSD-affected brain regions. The imaging findings and hyperbaric oxygen therapy effects indicate that PTSD can no longer be considered strictly a psychiatric disease.

## Introduction

This is a systematic review of the evidence for hyperbaric oxygen therapy (HBOT) treatment of post-traumatic stress disorder (PTSD) symptoms.

Until recently, PTSD was implicitly a psychiatric disorder that ensues from a trauma that is sufficiently disturbing to leave a person with residual emotional and behavioral sequelae. The essential feature of PTSD, as defined in the Diagnostic and Statistical Manual of Mental Disorders, Fifth Edition (DSM-5) of the American Psychiatric Association (1), is the development of characteristic symptoms following exposure to one or more traumatic events in which the individual is exposed to actual or threatened death, serious injury, or actual violence. Intrusive symptoms (nightmares and flashbacks) are the hallmark of PTSD, while avoidance, hypervigilance, cognitive and mood changes are seen in most cases. The symptoms, severity, and persistence vary widely for unknown reasons (2).

Epidemiological studies report that over half of the general population are exposed to a serious traumatic event in their lifetime, but only about 7 percent of those are likely to develop PTSD (3). However, among military personnel who have been involved in combat, the prevalence of PTSD is much higher and is often comorbid in 37% of servicemembers with mild traumatic brain injury (mTBI) (4).

Treatment of PTSD is problematic, especially for the persistent, disabling, or more severe forms. When PTSD becomes chronic nearly half of patients become treatment resistant (5, 6). Meta-analyses (7, 8) show that psychological interventions can significantly reduce PTSD symptoms when compared to control subjects; however, the effect sizes are often small or not reported. Pharmacological interventions were shown to be less effective than trauma-based psychological treatments for reducing PTSD symptoms or improving sleep with less than 60 percent reporting a meaningful clinical response and only 20 to 30 percent reporting remission (9, 10).

Improved brain imaging technology and research on U.S. war veterans from the Iraq and Afghanistan conflicts have revealed PTSD-associated changes in brain structure and function. This suggests that PTSD can no longer be considered a strictly psychiatric disease. Fear-conditioned learning was always identified as one of the main causative factors in PTSD. The growing literature, however, shows that PTSD is associated with specific neuroanatomical abnormalities or changes in fear neural pathways including the thalamus, amygdala, hippocampus, and medial prefrontal cortex (11–13). Veterans with severe PTSD show an indentation in the centromedial amygdala (14). A meta-analysis (15) reported a significant gray matter volume reduction of the left parahippocampal gyrus. Multiple studies demonstrate reductions in grey matter volumes in the amygdala, hippocampus, and prefrontal cortex, and the white matter volumes in the tracts connecting these three regions, including the cingulum bundle, uncinate fasciculus, and fornix/stria terminalis (16). Studies also reveal that PTSD is associated with a smaller hippocampus (17, 18). People with PTSD have been shown to have decreased activity in the prefrontal cortex which helps regulate the emotional responses triggered by the amygdala. It is not clear whether some neural circuit PTSD-associated abnormalities are present before a trauma or if the abnormalities develop after. Nonetheless, exposure to traumatic events has been shown to cause long-term changes in brain activity and in microstructure (19–21). The PTSD-related brain changes identified in these studies suggest wounding and inflammatory processes (19) that could be responsive to treatments for wounds and inflammation (22).

Hyperbaric oxygen therapy (HBOT) has been historically defined as intermittent treatment with 100% oxygen at a minimum arbitrary pressure of 1.4 atmospheres absolute (ATA) and more recently 1.0 ATA for a narrow list of acute and chronic wound conditions (22, 23). The U.S. Food and Drug Administration (FDA) corrected the confusion and misunderstanding in this definition by re-categorizing HBOT as a prescription medical drug (oxygen) and device consisting of increased barometric pressure and hyperoxia (24). Scientifically, it has been defined as “a medical treatment that uses increased atmospheric pressure and increased oxygen as drugs by fully enclosing a person or animal in a pressure vessel and then adjusting the dose of the drugs to treat pathophysiologic processes of the diseases” (25, 26). The exposure to increased atmospheric pressure and hyperoxia must be intermittent to achieve the therapeutic benefit, but the length and depth of exposure, use, frequency, and number of air breaks, frequency and total number of treatments, total oxygen and pressure dose, i.e., all variables of dosing, have not been well-defined. Based on this scientific definition, HBOT can be appreciated as a treatment for common acute and chronic wound pathophysiology (23, 25, 27, 28) found in acute and chronic wound conditions (23, 25, 27–30) and inflammatory conditions (28–33).

PTSD responsiveness to HBOT was first suggested as a serendipitous finding in the treatment of a U.S. war veteran with mTBI Persistent Postconcussion Syndrome (PPCS) (34). This positive response was replicated in a case series of 30 mTBI PPCS veterans (87% with PTSD) (35, 36), and a series of U.S. Department of Defense (DoD) sponsored civilian (37) and military-conducted studies (38–41). Due to misinterpretation of the DoD studies’ data based on design flaws and the confused definition of HBOT (22, 23), the efficacy of HBOT in mTBI PPCS as well as PTSD has been unclear. A recent systematic review and dosage analysis of these studies clarified the confusion (42). By analyzing both the combined and separate effects of increased barometric pressure and hyperoxia, according to the scientific and FDA’s understanding of HBOT, efficacy for HBOT in mTBI PPCS was demonstrated in a narrow pressure and wide oxygen dose range. Using the same analysis, this is a systematic review of the effectiveness of HBOT in the treatment of both military individuals and civilians with PTSD symptoms or PTSD.

## Methods

This systematic review was reported in accordance with the Preferred Reporting Items for Systematic Reviews and Meta-Analyses (PRISMA) guidelines (43). The PRISMA abstract and manuscript checklists are attached as Supplementary Files S1, S2.

### Search method

PubMed, CINAHL, and the Cochrane Systematic Review Database were searched without filters or time limits from 9-18-2023 to November 23, 2023 for English language clinical articles with HBOT treatment and PTSD symptom outcome data, using the search terms “hyperbaric oxygen” or “hyperbaric oxygen therapy” AND “stress disorder,” “posttraumatic stress disorder,” “post-traumatic stress disorder,” or “posttraumatic stress disorder, or “PTSD,” or “PTSD symptoms,” or “posttraumatic stress disorder symptoms.” Inclusion criteria were studies reporting pre-and post-HBOT treatment data, adult subjects 18–65 years old, civilian, or military, with and without a history of mTBI. The searched lists from each database were crosschecked against each other by visual inspection of titles to remove duplicates. Reference lists were reviewed for additional studies. Pooled studies, case studies, long-term follow-up, or reviews were excluded. PTSD outcome data had to be: Posttraumatic Stress Disorder CheckList – Military (PCL-M), Posttraumatic Stress Disorder CheckList – Civilian (PCL-C), Posttraumatic Stress Disorder CheckList – 5 (PCL-5), PTSD Symptom Scale Interview (PSS-I), or the Clinician Administered PTSD Scale (CAPS or CAPS – 5). The search process consisted of first search (title screen), second search (abstract review of first search titles), third search (full-text articles of abstracts), fourth search (detailed review of full-text articles).

### Demographics and data extraction

The demographic characteristics of the final selected articles are shown in Table 1. Numerical data includes date of publication, study design, number and sex of subjects, age, percentage diagnosed with PTSD, military or civilian, years of education and number of months from trauma to treatment with HBOT.

**Table 1:** Demographics of the analyzed studies.

Study	Design	Number Ss, Sex (M, F, U)	Age (yrs.)	% Dx’ed. w/PTSD	Military(AD), Vet (V), Civilian (C)	Education (yrs.)	Time from trauma to HBOT (mos.)
Wolf et al. (38)	RCT, DB	50 (48 M, 2F)	28.3	U	MI-AD	12+	3–71
Cifu et al. (39)	RCT, DB	61 (M)	23.2	U	MI-AD	U	8.5
Miller et al. (40)	RCT, DB	72 (69 M, 3F)	31.4	65	MI-AD	2/3^rd^’s with >12	22.9
Harch et al. (36)	Prospective Case Series	30 (28 M, 2F)	30.3	77	MI-(11 AD, 19 V)	13.1	40.2
Weaver et al. (41)	RCT, DB	71 (70 M, 1F)	32.8	49	MI-(68 AD, 3 V)	82% “some college”	25.6
Hadanny et al. (44)	RCT, SB C-O Control	30 (30F)	45.9	100	C	16.5	450
Harch et al. (37)	RCT, SB C-O Control	50 (21 M, 29F)	42.5	0	C-41, MI-9 (1-AD, 8-V)	14.0 (HBOT), 15.6 (Control)	55.2
Doenyas-Barak et al. (45)	RCT, SB	29 (U M, U F)	39 HBOT, 32C	100	MI-35 (all V)	14.2-HBOT, 13.7-C	138-HBOT, 133-C

RCT, randomized controlled trial; DB, double blinded; SB, single blinded; C-O Control, Cross-Over of the Control group to the Treatment; U, unstated, Military-AD, Military Active Duty; V, Veteran.

Data was extracted from each study and entered into Table 2. Numerical data include traditional pressure/oxygen dose parameters, length of time in each session, total number of HBOT’s in the protocol, outcome measure used in each study, number of points change pre to post within each group, percent change in PTSD symptoms for the designated outcome instrument, *p*-value, and whether the number of points change meets the criteria for Reliable Change (RC) or Clinically Meaningful Change (CMC). The total summative oxygen dose in excess of room air at 1 ATA for a course of treatment in each group of every study was calculated in atmosphere-minutes (AMs) according to the formula:

$$\begin{array}{l} AMs=\mathrm{Hyperbaric}\;\mathrm{Pressure}\;\left(ATA\right)\;x\;{FiO}_{2}\;x\;\\ \mathrm{Time}\;\mathrm{of}\;\mathrm{oxygen}\;\mathrm{exposure}\;\left(\mathrm{mins}.\right)\;x\;\mathrm{Number}\;\mathrm{of}\;\mathrm{HBOTs} \end{array}$$

**Table 2:** Change in PTSD symptoms by study, pressure and oxygen dose.

Study/Type Ss^a^	ATA x O^2^	Time/# HBOTs	O^2^ Dose (AM)	Measure^b^	Pre-Post Tx Change	% Change/p^c^/RC^d^
Wolf (38) / 1	2.4 × 100%	90 m / 30	6,900	1	50–41.6 = 8.4	−17% / 0.05 / RC
“	1.3 x air	90 m / 30	1,002	1	48.9–40.6 = 8.3	−17% / 0.05 / RC
Cifu (39) / 1	2.0 × 100%	60 m x 40	4,860	1	49.4–42.6 = 6.8	−14% / 0.05 / RC
“	2.0 × 75% (1.5)	60 m x 40	3,720	1	44.7–43.3 = 1.4	−3% / ns / ns
“	2.0 ×10.5%	60 m x 40	76	1	45.1–43.9 = 1.2	−3% / ns / ns
Miller (40) / 1	1.5 × 100%	50 m x 40	3,120	2	48.5–43.5 = 5	−10% / u / RC
“	1.2 x air	50 m x 40	600	2	53.5–42.1 = 11.4	−21% / u / CM
“	Routine Care	--	--	2	51.8–49.7 = 2.1	−4% / ns / ns
Harch (36) /1, 2	1.5 × 100%	50 m x 40	3,420	1, 3	63.4–46.8 = 16.6	−26% / 0.001 / CM
Weaver (41) / 1	1.5 × 100%	50 m x 40	3,120	2	54.9–45.6 = 9.3	−17% / u / RC
“	1.2 x air	50 m x 40	600	2	52.1–55.1 = +3	+6% / u / ns
Hadanny (44) / 4	2.0 × 100%	90 m x 60	11,400	3, 4, 6	28.9–20.7 = 8.2	−28% / 0.006 / u*
“	No Tx Control	--	--	3, 4, 6	29.9–28.4 = 1.5	−5% / ns / ns
“	2.0 × 100% C-O	90 m x 60	11,400	3, 4, 6	28.4–21.8 = 6.6	−23% / 0.005 / u*
Harch (37) /2,3,4	1.5 × 100%	50 m x 40	3,420	1, 2	37.9–26.0 = 11.9	−31% / 0.0001 / CM
“	No Tx Control	--	--	1, 2	39.7–37.5 = 2.2	−6% / ns / ns
“	1.5 × 100% C-O	50 m x 40	3,420	1, 2	39.7–29.2 = 10.5	−26% / 0.0001 / CM
D-Barak (45) / 2	2.0 × 100%	90 m x 60	11,400	5, 6	46.6–28.5 = 18.1	−39% / 0.001 / RC
“	Routine Care	--	--	5, 6	49.5–51.5 = +2.0	+4% / ns / ns

Negative values are a reduction in symptoms (improvement), positive values an increase (worsening) of symptoms.

a Type Subjects: 1 = Military Active Duty, 2 = Military Veterans, 3 = Civilians men, 4 = Civilians women.

b Measure 1 = PCL-M, Measure 2 = PCL-C, Measure 3 = SPECT, Measure 4 = PSS-I, Measure 5 = CAPS-5, Measure 6 = MRI, DTI, and fMRI.

c u = Unreported or Unknown information. ns = not significant.

d RC = Reliable Change; CM = Clinically Meaningful. Reliable change for CAPS-5 is >/= 13, For the PCL-M or PCL-C it is 5–10 and CM is >/= to 11 points. No Reliable Change information was found for the PSS-I but it is considered highly reliable and similar to the PCL.

FiO_2_ is fractional inspired oxygen percentage. The AMs were computed for every phase of a hyperbaric treatment: compression, time at depth, air breaks, and decompression. Constant compression and decompression rates were assumed. The average pressure from the surface to depth (compression) and depth to the surface (decompression) was multiplied by the FiO2 of the breathing gas for these phases. A linear increase in FiO2 and ∼90% FiO2 was assumed by 8 min of compression (46) for protocols in monoplace (single-person) chambers that compressed with 100% oxygen.

The selected studies used different instruments with different scales to measure the same PTSD symptoms (8, 47). To normalize and account for the differences between instruments the PTSD symptom outcomes were converted to percent change from baseline pre-randomization/treatment. The percent changes were averaged for each dose of pressure and summative oxygen and compared separately by hyperbaric treatment pressure (ATA) and the summative oxygen dose (AMs). The amount of symptom change was assessed for statistical significance and whether the change was sufficient to be considered a Reliable Change (RC) or a Clinically Meaningful Change (CMC), according to the guidelines developed by the National Center for PTSD. PTSD symptom changes of 5 to 10 points are considered an RC for the PCL. A 10-to-20-point change is a CMC (48). A change in the CAPS is equivalent to a 0.75–0.82 SD change of PCL (47). A within-person CAPS-5 change of 12 or more points is an RC (47). CAPS changes were considered equivalent to PSS-I changes due to high internal consistency, high interrater reliability, and no difference between the measures (8). All searches, screening, selection, data extraction, and analyses were performed by the two authors independently and then together. No automation tools were used.

### Methodologic quality and risk of bias assessment

The physiotherapy evidence database (PEDro) scale (49) was used to assess the methodologic quality/risk of bias of the randomized trials included in the final analysis and is presented in Table 3. Randomized trials are given a total score of 0–10 based on individual scoring of 10/11 items (the first, eligibility criteria, is omitted from the score because it is an external validity item), using 1 point for “present,” and 0 points for “absent.” The 10 items beginning with #2 are: (2) random allocation, (3) concealed allocation, (4) groups similar at baseline, (5) subject blinding, (6) therapist blinding, (7) assessor blinding, (8) 1 key outcome for >85% of subjects, (9) 1 key outcome: intention-to-treat analysis, (10) 1 key outcome between-group statistical comparison, and (11) 1 key outcome point measurements and variability. Scoring was performed by both authors based on stated scoring items in the text of each article and is presented in Table 3. Additional reporting bias and conflict of interest were noted for investigators who were employees of the funding source. All items were scored as 0 if they were not mentioned. Attempts were made to contact the authors of the studies to resolve the scoring on allocation concealment. Quality of studies was judged according to the scoring legend of Cashin et al. (50): poor (<4), fair (4, 5), good (6–8), and excellent (9, 10). Studies were independently scored by one author, reviewed by the other author and then conjointly scored by both authors.

**Table 3:** PEDro analysis of methodologic quality and risk of bias.

Items	1	2	3	4	5	6	7	8	9	10	11	Total
Wolf (38)	Y	Y	Y	Y	Y	N	Y	Y	Y	Y	Y	9
Cifu (39)	Y	Y	Y	Y	Y	N	Y	Y	Y	Y	Y	9
Miller (40)	Y	Y	Y	Y	N	N	Y	Y	Y	Y	Y	8
Weaver (41)	Y	Y	Y	Y	Y	N	Y	Y	Y	Y	Y	9
Harch (37)	Y	Y	Y	Y	N	N	Y	Y	Y	Y	Y	8
Hadanny (44)	Y	Y	Y	Y	N	N	Y	Y	Y	Y	Y	8
D-Barak (45)	Y	Y	N	Y	N	N	Y	Y	Y	Y	Y	7

## Results

**Figure 1:**
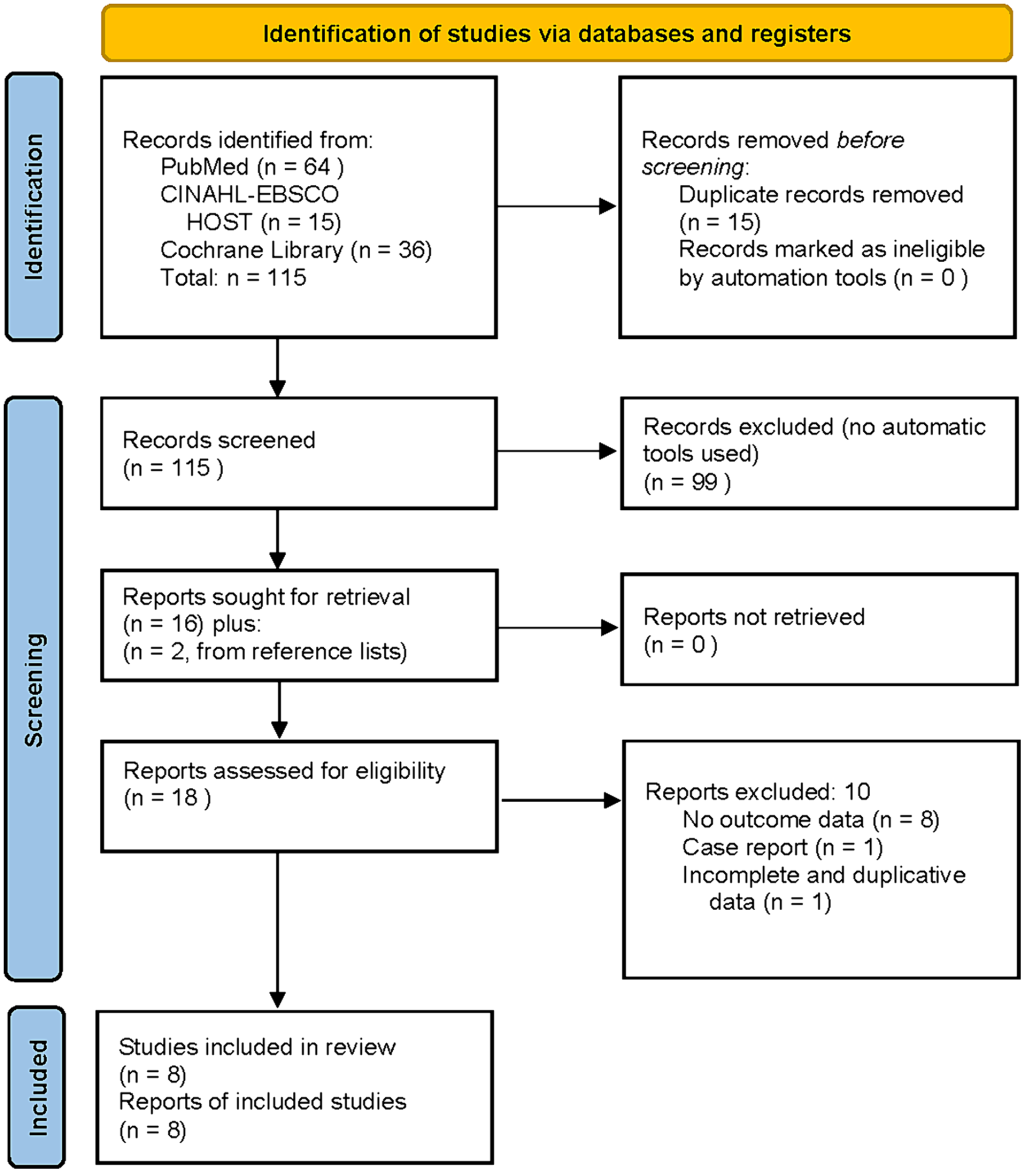
Literature search: hyperbaric oxygen therapy treatment of post-traumatic stress disorder.

Literature search yielded 115 articles [PRISMA flow chart (43), (Figure 1)]. Eight studies met inclusion criteria, were included in the final analysis, and are listed chronologically in Table 1. Four of the studies in Table 1, (38–41), were U.S. Department of Defense (DoD) sponsored. The subjects were active duty military or veterans from the Middle East conflicts with comorbid mTBI. Some percentage of all four studies’ subjects had comorbid diagnoses of PTSD. All four studies were designed to compare the effects of hyperbaric oxygen therapy on mTBI. PTSD was a secondary measure using the PCL-M or PCL-C administered before and after treatment. All four studies also attempted to control the placebo effect of the hyperbaric exposure with purported sham exposures. These sham exposure groups were based on the historical misdefinition of HBOT (22, 23) that defined HBOT as the use of 100% oxygen at ≥1.4 ATA. The bioactive components of HBOT are increased barometric pressure and hyperoxia (25, 26, 42). A sham treatment must omit both of these to control for the independent effects of pressure and hyperoxia. By design none of the sham groups in the 4 DoD studies could do this. The sham exposures are pseudo-sham/pseudo-control groups that used lower doses of hyperbaric oxygen therapy as a treatment. Thus, they are analyzed as comparative dosing studies. The only DoD study with an acceptable control group (40) used a no-HBOT treatment control group, similar to the civilian studies (37, 44, 45).

Wolf et al. (38), the first of the DoD studies, is a comparative dosing study that randomized 50 active-duty military, to treatment and “control” groups for thirty 90-min exposures to 2.4 ATA 100% oxygen (2.4 ATA oxygen, 6,900 AMs oxygen) or 1.3 ATA 21% oxygen, room air (0.27 ATA oxygen, 1.002 AMs oxygen). Within-group change in PCL-M symptoms was a statistically significant 8.4-point RC decrease in PTSD symptoms for the 2.4 ATA group (50–41.6, *p* < 0.05) and a near identical significant RC decrease of 8.3 points (48.9–40.6, p < 0.05) for the 1.3 ATA 21% oxygen group. No statistical analysis of change scores between the two groups was performed. No conclusions were drawn about the efficacy of HBOT for PTSD.

Cifu et al. (39) was a similar comparative dosing study of three different doses of oxygen at a constant pressure. The study randomized sixty-one male military subjects to forty 60-min exposures to 2.0 ATA 100% oxygen (2.0 ATA oxygen, 4,860 AMs oxygen), 2.0 ATA 75% oxygen (1.5 ATA oxygen, 3,720 AMs), or 2.0 ATA 10.5% oxygen (0.21 ATA oxygen, 76 AMs). There were no differences in PCL-M scores between-groups before treatment or between groups after treatment, but no change score differences between-groups were analyzed. Within group analyses showed a statistically significant RC score PCL-M improvement of 6.8 points (49.4 to 42.6, *p* < 0.05) for the 2.0 ATA 100% oxygen dose group and non-significant change scores of 1.4 points (44.7 to 43.3) for the 1.5 ATA oxygen group and 1.2 points (45.1 to 43.9) for the 0.21 ATA oxygen group. No conclusions were drawn about the efficacy of HBOT for PTSD.

Miller et al. (40) is the only controlled DoD study. It randomized 72 subjects to forty 60-min exposures to 1.5 ATA 100% oxygen (1.5 ATA oxygen, 3,120 AMs), 1.2 ATA 21% oxygen, room air (0.25 ATA oxygen, 600 AMs), or a true control group of Routine Care without hyperbaric treatment. Within-group treatment effects showed a CMC 11.4 point PCL-C decrease (53.5–42.1) in the 1.2 ATA group, a 5.0 point RC decrease in the 1.5 ATA group (48.5–43.5), and a 2.1 point decrease (51.8–49.7) in the Routine Care group which was neither aCMC nor an RC. There were no between group, within group, or treatment vs. true control group statistical analyses performed. The study noted improvement in PTSD symptoms “after the interventions, favoring the “sham” group… over the HBO group…,” but no conclusions were drawn about the efficacy of HBOT for PTSD.

The fourth DoD study, Weaver et al. (41) randomized 71 mostly active duty military members with mTBI PPCS to the same HBOT treatment schedule as Miller et al. (40), but did not include a true no-treatment control group. Subjects received forty 60 min exposures to 1.5 ATA 100% oxygen (1.5 ATA oxygen, 3,120 AMs) or 1.2 ATA 21% oxygen, room air (0.25 ATA oxygen, 600 AMs). Between-group analysis demonstrated a statistically significant change score difference between groups (improvement in favor of the 1.5 ATA oxygen group) of 7.3 points (−13.5, −1.0; *p* = 0.02), despite a significant disease severity bias against the 1.5 oxygen group. The 1.5 ATA oxygen group was older, had more combat deployments, worse anger control, and higher mean pre-treatment PCL-C scores than the 1.2 ATA group. When those subjects diagnosed with PTSD by the Structured Clinical Intervidew for DSM-IV PTSD module (the PTSD subgroups) were analyzed the 1.5 ATA oxygen HBOT benefit was even greater: a 12.3 point statistically significant (−21.4, −3.1; *p* = 0.01) difference between groups. This was composed of a 9.3 point RC improvement for the 1.5 ATA oxygen group and a 3.0 point worsening of the 1.2 ATA (0.25 ATA oxygen) group.

Summarizing the above four studies, RC improvements in PTSD symptoms have been demonstrated at 2.4 ATA oxygen and 1.3 ATA air (0.27 ATA oxygen) (38), 2.0 ATA oxygen (39), 1.5 ATA oxygen (40, 41), and CMC at 1.2 ATA air (0.25 ATA oxygen) (40). The Wolf et al. (38) and Cifu et al. (39) dose group improvements were statistically significant within-group changes and the Weaver et al. (41) study showed a statistically significant between group improvement in favor of the 1.5 ATA oxygen group compared to the 1.2 ATA air group. In the only study with a true control group, Miller et al. (40), the CMCs and RCs within-groups occurred only in the two treatment groups while none occurred in the No Treatment control group. The remainder of the within group changes (40), between group changes (38, 39), and changes compared to true control group (40) were not statistically analyed in the studies.

The remaining 4 studies consisted of a case series with imaging controls and three studies with true treatment controls. The U.S. DoD-administrated case series of Harch et al. (36) prospectively treated 30 active duty and retired military members with mTBI PPCS, 23 of whom met the PCL-M threshold for PTSD diagnosis. The subjects received forty 60-min exposures to 1.5 ATA 100% oxygen (1.5 ATA oxygen, 3,420 AMs oxygen). The subjects had the greatest pre-treatment PTSD scores (mean of 63.4) of all the studies with military subjects, which was 32% higher than subjects in the four DoD studies. These subjects experienced the greatest reduction in the PCL, 16.6 points. This was statistically significant (−22.6, −10.6; *p* < 0.001) and a CMC. The controlled neuroimaging component of the study demonstrated significant SPECT brain blood flow improvements in right posterior hemispheric gray and white matter regions in the HBOT-treated veterans compared to controls.

Two of the three remaining studies, Hadanny et al. (44) and Harch et al. (37), used a randomized crossover design where all subjects received HBOT and the randomly assigned control group crossed over to receive HBOT after the control period. Hadanny et al. (44) is the only study that was exclusively non-military subjects, all women (with sexual trauma in childhood), and had no comorbid TBI. Thirty women received sixty 90-min exposures to 2.0 ATA 100% oxygen (2.0 ATA oxygen, 11,400 AMs). The treatment group experienced a statistically significant 8.2 point (28.9–20.7, *p* = 0.006) decrease in the PSS-I compared to 1.5 points in the control group (29.9–28.4, *p* = 0.22). After the control period the control group crossed-over and was treated with the same 2.0 ATA 100% oxygen, 11,400 AMs. Following treatment, the crossed-over control group experienced a significant 6.6 point (28.4 to 21.8, *p* = 0.005) decrease in PTSD symptoms. No statistical analysis of change scores between groups was performed. The raw point reductions would be reliable changes on a PCL questionnaire. Considering the condensed PSS-I scale vs. the PCLs (51 points vs. 85 points) if re-scaled to a PCL questionnaire the scores for CMC and RC would be 14.5 and 11.0 points, respectively. SPECT brian blood flow imaging analysis revealed significant increases in blood flow in 7 Brodmann areas compared to the control group. Correlation analysis of perfusion changes and questionnaire scores were significant between 7 Brodmann areas and multiple questionnaire components. MRI DTI results demonstrated significant increases in fractional anisotropy (FA) in anterior thalamic radiation, left insula, right thalamus, and superior thalamic radiation (*p* < 0.001) in HBOT subjects compared to controls. When all HBOT-treated subjects (HBOT group and crossover Control Group) were compared to Controls pre-HBOT three of the four significant findings persisted; superior thalamic radiation was no longer significant.

The second crossover study, Harch et al. (37), was a study on mTBI PPCS that excluded subjects with CAPS scores high enough to be diagnosed as PTSD. Sixty-three subjects, 83% civilian and 17% military, were randomized to forty 60-min exposures to 1.5 ATA 100% oxygen (1.5 ATA oxygen, 3,420 AMs) or a true control group without hyperbaric treatment. The Control group then crossed over to receive the same 1.5 ATA oxygen HBOT treatment at the end of the control period. Between-group analysis of PCL change scores demonstrated a statistically significant change score difference (improvement in favor of the 1.5 ATA oxygen group) of 9.7 points (−17.7, −8.6; *p* = 0.0001). Within-group analyses showed a statistically and CMC score improvement of 11.9 points (37.9–26.0, *p* < 0.0001) for the 1.5 ATA oxygen dose group and non-significant change scores of 2.2 points (39.7–37.5, p = n.s.) for the no treatment control group. After the Controls crossed over and received HBOT treatment they achieved a similar statistically significant and CMC decrease in PCL-C of 10.5 points (37.5–27.0, *p* < 0.0001).

The final study by Doenyas-Barak et al. (45) was similar to Hadanny et al. (44). Both studied treatment-resistant PTSD without comorbid TBI using identical doses of HBOT, but Doenyas-Barak et al. (45) studied military members in a treatment vs. true control no-treatment design, while Hadanny et al. (44) studied civilians in a crossover true control design. Doenyas-Barak et al. (45) randomized 28 veterans to sixty 90-min exposures to 2.0 ATA 100% oxygen (2.0 ATA oxygen, 11,400 AMs) or a control group without HBOT treatment who received their ongoing psychotherapy. Within-group analyses demonstrated a statistically and clinically significant decrease (improvement) in CAPS-5 score of 18.1 points in the HBOT group (46.6–28.5, C.I: −25.4, −10.8, *p* < 0.0001) compared to a 2.0 point increase (worsening) in the control group (49.5–51.5, *p* = 0.211). Cohen’s net effect size was 1.643. Translating the CAPS to a PCL score indicates that this is a 25.9 point decrease in the PCL, a very large change score. Brain imaging MRI DTI demonstrated significant increases in fractional anisotropy in the left anterior and posterior limbs of the internal capsule and right parietal white matter in HBOT-treated subjects compared to controls. Functional MRI showed within-group increases in fMRI BOLD signal for the HBOT-treated subjects in the left dorso-lateral prefrontal, middle temporal and temporal gyri, both thalami, left hippocampus, and left insula. No significant within group changes were found for the control group. Statistically significant correlations were found between mean percent BOLD signal changes in peak significantly activated regions and percent change in total CAPS score (r = 0.42–0.67, *p* < 0.05).

In summary, six of the eight studies were conducted on military subjects and two on civilians. Two studies were designed to investigate PTSD without TBI. One of these studies was on military subjects with treatment resistant PTSD who did not have a TBI and the other involved women who were sexually abused as children with long-term PTSD. There are four studies with five pseudo-sham control groups that received lower doses of hyperbaric oxygen therapy. Two of the five pseudo-sham groups showed significant improvement in PTSD, one at 1.3 ATA and the other at 1.2 ATA. The other 3 pseudo-sham treatments only showed −1 point (improvement) to +3 points (deterioration).

### Dose analysis of studies

**Figure 2:**
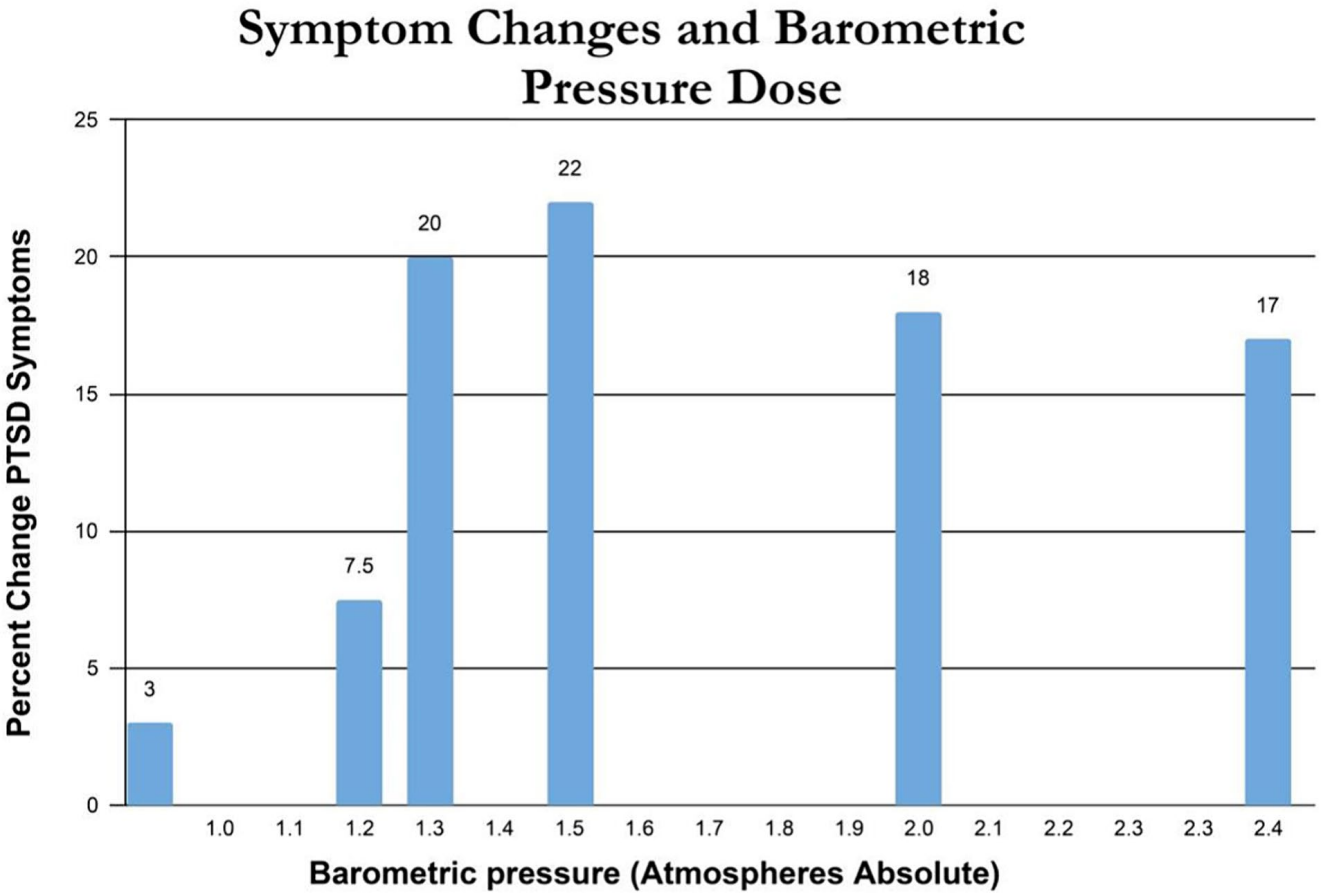
Symptom changes and barometric pressure dose.

**Figure 3:**
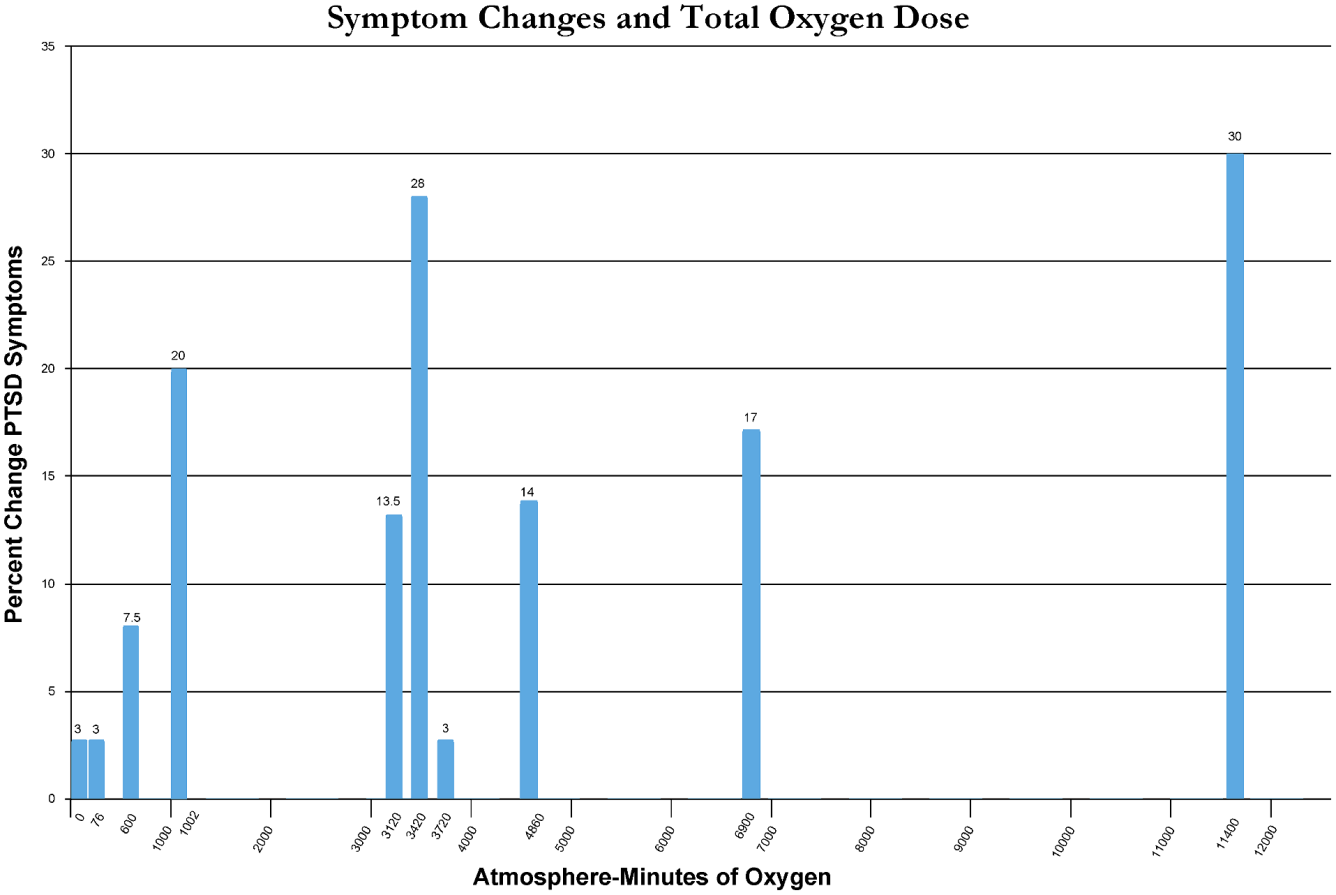
Symptom changes and total oxygen Dose.

Percent change in immediate post-treatment or control PTSD symptoms for each study’s groups is presented in Table 2. These percent changes are further compared by treatment pressure (Figure 2) and total oxygen dose from the repetitive intermittent exposures to hyperoxia (Figure 3). Figure 2 shows a minimum pressure threshold for improvement in PTSD symptoms at 1.3 ATA that is relatively constant for increased pressures upto 2.4 ATA. Figure 3 shows a minimum total oxygen dose threshold for improvement in PTSD symptoms of 1,002 AMs (20% improvement) and varied improvements in symptoms with greater oxygen doses upto 11,400 AMs (13.5–30%). The one outlier figure of 3,720 AMs (3% improvement) in this range of AMs greater than 1,002 AMS was the only mixed dose of oxygen and pressure where oxygen was not 100% of the total pressure dose (1.5 ATA oxygen, 2 ATA pressure).

**Figure 4:**
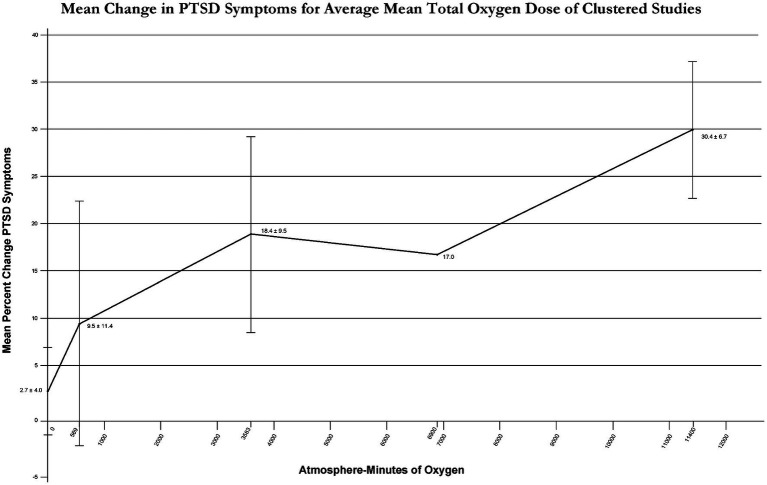
Mean change in PTSD Symptoms for average mean total oxygen dose of clustered studies.

To clarify the effect of total oxygen dose on outcome from intermittent exposures to hyperoxia Figure 3 outcomes were graphed with average % improvement in PTSD symptoms for narrow ranges of total oxygen dose (Figure 4). Figure 4 demonstrates a near-linear dose–response curve for increasing PTSD symptom improvement with increasing total oxygen dose.

### Methodologic quality, and risk of bias assessment

According to PEDro scoring statistics (51), studies scoring ≥6/10 on the PEDro scale are considered “moderate to high quality.” Table 3 presents the PEDro analysis for the seven randomized studies reviewed. All seven scored studies met criteria for moderate to high quality. Using the qualitative assessment of Cashin et al. (50) for the entire scale of PEDro Scale scores, all four randomized controlled trials (37, 40, 44, 45) and all three randomized non-controlled studies were considered good to excellent quality/bias with PEDro scores of 8 (40), 8 (49), 8 (37), 9 (38), 9 (39), 9 (41), and 7 (50). Despite the methodological rigor of these hyperbaric studies, PEDro scores are compromised by 1–2 points each due to the inherent blinding problems of hyperbaric treatment, similar to surgical and other human performed therapies. Chamber operators (therapists) cannot be blinded to treatment group in any of the studies (1 point) and subjects are not blinded in any no-treatment control group study (1 point), especially the crossover studies. An additional source of bias not addressed in the PEDro analysis is bias from conflict of interest. Dr. Harch stated conflict of interest in his studies (36, 37). Many of the investigators in the other studies are employed by the funding source (38–41, 45) or have another conflict of interest (51).

### Side effects

Side effects and adverse events are listed in Table 4 and were generally minor and transient. Mild reversible middle ear barotrauma (MEBT), the most common complication seen in HBOT studies, occurred in 5.5–43% of the subjects. This range was higher than historical figures of 2% (23), the incidence in Harch et al. (37). Some of the higher figures were explained by chamber operations/operators/equipment at one study site (41) and intensity of schedule (twice/day treatments) with more frequent upper respiratory infections (36). The high rate was not explained in the other studies (44, 45). Total side effects and adverse events were combined and reported in Churchill, et al. (52) for Weaver et al. (41) and Miller et al. (40) at 33% (40/120), 77% of which were due to otic, sinus, and tooth barotrauma. Total complication rate in Harch et al. (37) was 8%.

**Table 4:** Side effects and complications.

Study	Middle ear barotrauma	Other minor side effects	Significant adverse events
Wolf (38)	5.5%	0.07–0.61%: sinus squeeze, confinement anxiety, headache, nausea, numbness, heartburn, musculoskeletal chest pain, latex allergy, and hypertension	
Cifu (39)	None reported	None reported	None reported
Miller (40)	Reported combined with Weaver, *et al*	Reported combined with Weaver, *et al*	Reported combined with Weaver, *et al*
Harch (36)	20%	23%: transient worsening of TBI/PTSD symptoms at midway through treatment	6.7%: transient exacerbation of anxiety
Weaver (41)	17%	8.3%: sinus barotrauma 0.8%: tooth barotrauma. 6.7%: headache, 2.5%:dizziness/vertigo 2.5%: vision change, 1.6% each: anxiety and somnolence, 0.8% each: dyspnea, neck irritation, eye pruritis, or hyperventilation	
Hadanny (44)	43%	3.6%: headache	32%: reversible emotional flooding during 1^st^ 20 HBOTs
Harch (37)	2%	1%: perforated tympanic membrane, 4%: late protocol fatigue	
Doenyas-Barak (45)	39%		39%: reversible surfacing of new memories/severe distress

Three of the studies reported an unusual side effect, worsening of emotional symptoms transiently during treatment that occurred in at least 30% of subjects. Harch et al. (37) noted exacerbation of PTSD anxiety (2/30 subjects, 6.7%), and transient worsening of some TBI/PTSD symptoms at the midway point (7/30 subjects, 23%), while Hadanny et al. (44) documented 32% (9/28 subjects) with “emotional flooding” (re-experiencing memories of childhood event) during the first 20 HBOTs, and Doenyas-Barak et al. (45) reported a similar 39% (7/18 subjects) with “unexpected surfacing of new memories…” that “surfaced gradually, during the second half of the treatment course (after 25–35 sessions of HBOT).” Some of these three studies’ events appear to be serious adverse events. Two of Harch et al.’s (37) patients required emergency department visits, one of which re-instituted benzodiazepine medication that had been discontinued before the event that occurred during alcohol intoxication, and the second for evaluation of anxiety-induced chest pain/gastrointestinal distress. Hadanny et al. (44) does not elaborate on the severity of the emotional flooding. Doenyas-Barak et al. (45) stated that the recovery of the memories was accompanied by severe distress with “…resolution of the distress.” Despite this exacerbation of emotional symptoms in the studies all of the subjects were able to complete the treatment.

## Discussion

In this systematic review of HBOT treatment of PTSD symptoms eight studies (7 randomized trials) were analyzed for symptom outcomes according to composite doses of hyperbaric oxygen therapy and component doses of barometric pressure and hyperoxia. Overall, the studies’ data demonstrate that HBOT caused statistically significant, reliable change, or clinically meaningful change/improvements in PTSD symptoms and/or PTSD compared to true control groups and within nearly all treatment groups. In two of the studies with the greatest improvement symptom relief was obtained for treatment-resistant PTSD that was over a decade long. The improvements in three of the studies were supported by functional and anatomic imaging changes in PTSD-associated brain structures. These results were achieved despite heterogeneity of study design, statistical analysis, and subject populations. Treatment effects were non-specific to cause of PTSD, occurring in male and female subjects, both military and civilian, with childhood sexual trauma and fibromyalgia.

The symptom improvements were achieved with multiple different doses of HBOT and occurred at composite doses of oxygen and pressure at 1.3, 1.5, 2.0, and 2.4 atmospheres. The pressure threshold for improvement appeared to be 1.2–1.3 ATA and the total oxygen threshold of 1,002 atmosphere minutes. There was increasing benefit with increasing total doses of oxygen from 1,002 to 11,400 AMs. These total doses of oxygen are summative from 30 to 60 repetitive intermittent exposures to hyperoxia and pressure. The average greatest symptom improvement occurred with the highest total oxygen dose in two studies (44, 45), but the dominant contributing component(s) to this dose (FiO2, absolute pressure, length of each treatment, absence/presence/number of air breaks, treatment frequency, and total number of treatments) could not be identified. It may have been the presence of all of these variables in the regimen of these two studies that is responsible for the large symptom improvement, however a near equivalent percent symptom improvement occurred in Harch et al. (36) in similar subjects (44) with the same FiO2, half the increase in barometric pressure, half the treatment length, no air breaks, twice the frequency, two-thirds the number of treatments, and with a lesser incidence of emotional flooding/side effects. Due to the small number of studies and large number of variables it is impossible to draw further conclusions beyond increasing symptom improvement with increasing total oxygen dose. There is no evidence, however, that the HBOT-induced effects on PTSD could occur from a continuous total exposure to oxygen of 1,002–11,400 AMs.

All seven randomized trials were judged to be of moderate to high quality by the PEDro scoring system. The rigor of all seven studies was excellent, but due to limitations of blinding inherent to hyperbaric oxygen treatment their PEDro bias ratings were compromised by at least one point and in four of the seven by two points. PEDro criteria require blinding of subjects and all “therapists.” To blind subjects to both pressure and oxygen they must not go in a hyperbaric chamber. A no-chamber experience group unblinds the control subjects [one point PEDro scale loss each for four of the studies (37, 40, 44, 45)]. To blind all therapists, which includes chamber operators/gas composition allocators, the operators/allocators cannot know the pressures or gas compositions. Using the most sophisticated chamber designs and equipment, this is nearly impossible to achieve and was not achieved in the DoD studies (38–41): one-point PEDro scale loss each.

The inability to blind subjects and operator therapists to treatment group should predispose to placebo effects that lower the rigor and increase the bias of a study. It appears that these PEDro scale losses did not compromise the rigor of the studies because the placebo effect in these studies was negated by the pressure and oxygen dose analyses. The analyses showed that there are minimum thresholds necessary for treatment effects. If placebo was involved the treatment effect should occur regardless of oxygen or pressure dose. That did not occur. In addition, different effects were seen with different doses which is contrary to a placebo contribution. The placebo contribution is also negated by the Cifu et al. study (39) in which all subjects had the identical chamber experience yet only one group showed a significant outcome. If the ritual of the chamber experience had a placebo effect all of Cifu et al.’s (39) groups should demonstrate the same placebo outcome. They did not. Essentially, the PEDro scale overestimated the bias in the studies.

The most common side effect seen in HBOT, middle ear barotrauma, occurred in 5.5–43% of subjects. The higher range of this side effect in some of the studies was due to intensity of schedule in one study (37) and chamber operations in another study (41). In two other studies with the highest rates (44, 45) this was not explained. Other side effects were minor and/or rare. An unusual side effect that was documented in several studies was a significant, but reversible, revisiting of the old traumatic memories at the highest oxygen doses in 30–39% of subjects (44, 45). The surfacing of old traumatic memories is also considered to be part of the emotional healing process of PTSD, particularly when psychotherapy is provided at the same time. The investigators in these two studies suggest revisiting these memories is due to HBOT-induced metabolic and circuitry changes in brain areas associated with emotional and pain processing (44) and induced neuroplasticity effects in the hippocampus (45). Alternatively, this phenomenon may partly reflect increasing oxidative stress. The resurfacing of old memories was minor in the lower oxygen dose studies (36, 37), but more intense with the highest doses of oxygen, suggesting accumulating oxidative stress/toxicity from the intermittent exposure (42), despite air breaks. In the military study of Doenyas-Barak et al. (45) it occurred gradually with increasing number of treatments >25–35 HBOTs (2.0/90): 2 × 90 × 35 = 6,300 AMs, which is the similar point at which reversal of improvement in PTSD and mTBI PPCS symptoms occurred in the military study of Wolf et al. (38): 30 HBOTs at 2.4/90: 2.4 × 90 × 30 = 6,480 AMs. The Wolf et al. (38) effect at 2.4 ATA of oxygen has been identified as an oxidative stress/toxicity phenomenon (42). Regardless of the intensity, these side effects resolved during the completion of the therapeutic process.

PTSD is a mental health condition triggered by a terrifying event that causes flashbacks, nightmares, and severe anxiety. Once considered primarily a psychiatric disorder, PTSD is now understood to be accompanied by significant changes in the brain (11–21). These neuroanatomical changes help explain the behavioral changes and symptoms as the brain controls our behavior. However, more than explaining the behavioral changes of a person suffering from moderate to severe PTSD symptoms, the brain changes may also help us understand why almost 50% of PTSD sufferers fail traditional treatments and their PTSD becomes chronic and resistant to treatment. Many studies have demonstrated the brain changes in PTSD subjects (11–21); however, this is the first review of studies that measured pre-and-post HBOT brain changes.

Three of the studies in this systematic review (35, 36, 44, 45) included neuroimaging to measure brain changes in chronic and/or treatment resistant subjects as a function of the HBOT treatment. Harch et al. (36) demonstrated significantly increased SPECT brain blood flow in the right posterior hemispheric gray and white matter areas in the HBOT treated veterans compared to controls. Hadanny et al. (44) also included SPECT imaging and the brain blood flow imaging analysis shows significant increases in blood flow in 7 Brodmann areas compared to the control group before cross-over. Hadanny et al. (44) reported MRI DTI results of significant increases in FA in anterior thalamic radiation, left insula, and right thalamus compared to the control subjects pre-cross-over. The significant increases in FA persisted after all control subjects were treated. It is relevant that these women had chronic PTSD that went back to their childhood abuse, but none of the subjects had a history of brain trauma, such as mTBI.

The third study, Doenyas-Barak et al. (45), also studied treatment-resistant PTSD in military veterans with no history of TBI. The study included MRI DTI and demonstrated statistically significant increases in FA in the left anterior and posterior limbs of the internal capsule and right parietal white matter in subjects who received HBOT treatment versus the control subjects. Subjects also showed within-group increases in the fMRI BOLD, which signals an over-oxygenation (actively actuated increase in blood flow and volume) (53) for the HBOT subjects in the left dorso-lateral prefrontal, middle temporal, and temporal gyri, both thalami, left hippocampus and left insula. The increases in fMRI BOLD are usually regarded as increased/improved activity due to flow-metabolism coupling. No within group changes were found for the No Treatment Control subjects.

Hyperbaric oxygen therapy is a dual-component drug therapy consisting of intermittent increased barometric pressure and hyperoxia (25, 26) that has wide-ranging beneficial effects on acute and chronic wound pathophysiology (23, 25, 27, 28) found in acute and chronic wound conditions (23, 25, 27–30) and inflammatory conditions (28–33). HBOT has been demonstrated to have wide-ranging effects on inflammation and a dysregulated immune system (54–59) that may be due to its broad effects on expression and suppression of immune-active genes (60–62). The anatomic and functional imaging findings (11–21) and immune dysregulation in PTSD (19, 63) imply that the experience of a terrifying traumatic event triggers wounding and inflammatory changes in the brain. The structural and functional imaging changes documented in the reviewed studies (35, 36, 44, 45) suggest that the HBOT-generated improvement in PTSD symptoms are the result of HBOT’s well-documented effects on wound pathophysiology and wound-healing. This is apparent in the improvements in connectivity and blood flow in the hippocampus documented by Harch et al. (35) and Doenyas-Barak (45). Given the known immune system-activity of HBOT it is also likely that the symptomatic improvements are in part due to HBOT’s beneficial impact on the immune system dysregulation in PTSD. These imaging and immune system findings coupled with HBOT’s effects on inflammation and wounds suggests that PTSD can no longer be considered strictly a psychiatric condition.

### Summary of main findings

This systematic review demonstrated statistically significant, clinically meaningful, or reliable change improvements in multiple high quality low bias randomized controlled and randomized trials of HBOT treatment of subjects with PTSD or PTSD symptoms. Minimum thresholds of pressure and total hyperoxic dose were required for the improvements, but occurred across a broad range of pressures and total oxygen doses with a dose–response relationship of symptom relief to total oxygen dose. The greatest symptom relief was achieved at the highest doses, but were accompanied by a side effect of severe emotional distress in upto 39% of subjects that was reversible. Based on these studies’ data and this systematic review HBOT should be recommended as a treatment option for PTSD or patients with PTSD symptoms.

### Limitations

Possible minor limitations include the limited number of studies, PTSD as a secondary outcome in many of the studies, and the majority of the studies in military subjects. The minimal bias of the studies and uniformity of results within the structure of a systematic review, according to pressure and oxygen dose and regardless of military or civilian status, cause of PTSD, or sex of subjects, suggests that these are not significant limitations. The apparent major limitation of this review is the heterogeneity of the studies: designs, doses of hyperbaric therapy, dosing parameters, subjects, diagnoses (PTSD symptoms or PTSD, with or without comorbid TBI or fibromyalgia), and statistical analyses. Some were randomized trials comparing different doses of hyperbaric therapy, some had true no-treatment control groups, there was a wide range of pressures and total oxygen doses, presence or absence of air breaks, many of the studies consisted of predominantly males, one was all female, and some studies analyzed only within group treatment effects while others looked at pre-and post-treatment effects or between group change effects. This apparent limitation could be interpreted as a strength of the study and its conclusions. The PTSD population is a heterogenous population and the majority of the studies in this review involved comorbid mTBI PPCS patients which are a very heterogeneous group by nature of their comorbid diagnosis. This would normally argue for very large studies to neutralize the effects of so many variables. However, each of the limitations applied to only some of the studies and were balanced by similar treatment results in other studies without the limitations. Each of the limitations would be expected to compromise uniformity of results, but despite the heterogeneity the improvement in symptoms was substantial and when analyzed by dose shown to be fairly uniform above certain pressure and total oxygen thresholds, contrary to placebo effects.

Another apparent traditional limitation is the small sample size of the studies. Large, randomized trials are preferred for systematic reviews and evidence grading, however, small sample size as a contributor to a Type I error is often the result of imbalance of treatment groups for key variables that affect the outcomes. None of the studies featured unbalanced groups except Weaver et al. (41) where there was bias against treatment effect in the more severely affected HBOT group. Multiple Type I errors to explain multiple small trial positive results is unlikely. In addition, small sample size is usually a criticism of studies with negative outcomes due to a Type II error. The analyzed studies had positive outcomes. Overcoming a small sample size Type II error occurs when the treatment effect is large. The treatment effect was large in nearly all of the studies with CMC and RC. This supports the positive outcomes and conclusions of this systematic review.

### Implications for future research

This systematic review sheds new light on PTSD and its treatment. It implies that HBOT may be the treatment of choice when pathological brain changes exist, such as in two of the studies in this review with complex treatment-resistant PTSD (44, 45). New research could focus on identifying those PTSD patients with pathological brain changes and which elements of the trauma-PTSD pathogenesis predispose to brain pathological changes: the intensity or duration of the trauma experience, the duration of the PTSD, treatment resistance, or other factors. It is possible that milder or shorter-term reactions to trauma (PTSD symptoms without the diagnosis of PTSD) may not have permanent brain changes, however, it appeared from the Harch et al. RCT (37) where the diagnosis of PTSD was excluded that milder or shorter-term reactions to trauma may also be responsive to HBOT. Future research should be directed to comparative effectiveness studies of HBOT versus standard therapies in these milder cases. Another research focus would be the identification of biomarkers or behavioral markers of pathological brain changes that would avoid costly neuroimaging. A possible candidate is microRNA. MicroRNA has been successfully investigated in acute mTBI with (64) or without (65, 66) balance, cognitive testing, and symptoms.

## Conclusion

Multiple high quality low bias randomized controlled trials and randomized trials of HBOT in the treatment of PTSD symptoms or PTSD demonstrated statistically significant, clinically significant, and/or reliable change improvements in symptoms that occurred across a wide range of oxygen and pressure doses with a dose–response relationship for symptom relief and total oxygen dose. Side effects were minor except for a transient period of significant emotional symptoms at the highest oxygen doses.

## Data availability statement

The original contributions presented in the study are included in the article/Supplementary material, further inquiries can be directed to the corresponding author.

## Author contributions

SA: Conceptualization, Data curation, Formal analysis, Investigation, Methodology, Writing – original draft, Writing – review & editing. PH: Conceptualization, Data curation, Formal analysis, Funding acquisition, Investigation, Methodology, Project administration, Resources, Software, Supervision, Validation, Visualization, Writing – original draft, Writing – review & editing.

## Supplementary material

SUPPLEMENTARY FILE S1 PRISMA abstract checklist.

SUPPLEMENTARY FILE S2 PRISMA checklist.
